# Actin-rich lamellipodia-like protrusions contribute to the integrity of epithelial cell–cell junctions

**DOI:** 10.1016/j.jbc.2023.104571

**Published:** 2023-03-03

**Authors:** Yosuke Senju, Toiba Mushtaq, Helena Vihinen, Aki Manninen, Juha Saarikangas, Katharina Ven, Ulrike Engel, Markku Varjosalo, Eija Jokitalo, Pekka Lappalainen

**Affiliations:** 1 Helsinki Institute of Life Science (HiLIFE) - Institute of Biotechnology, University of Helsinki, Helsinki, Finland; 2 Research Institute for Interdisciplinary Science (RIIS), Okayama University, Okayama, Japan; 3 Faculty of Biochemistry and Molecular Medicine, Biocenter Oulu, University of Oulu, Oulu, Finland; 4 Helsinki Institute of Life Science (HiLIFE), University of Helsinki, Helsinki, Finland; 5 Molecular and Integrative Biosciences Research Programme, Faculty of Biological and Environmental Sciences, Neuroscience Center, University of Helsinki, Helsinki, Finland; 6 Nikon Imaging Center and Centre for Organismal Studies, Heidelberg University, Heidelberg, Germany

**Keywords:** adherens junction, actin, Arp2/3 complex, WAVE complex, membrane curvature, BAR domain, cell compartmentalization, epithelial cell, lipid-protein interaction, protein self-assembly

## Abstract

Metastasis-suppressor 1 (MTSS1) is a membrane-interacting scaffolding protein that regulates the integrity of epithelial cell–cell junctions and functions as a tumor suppressor in a wide range of carcinomas. MTSS1 binds phosphoinositide-rich membranes through its I-BAR domain and is capable of sensing and generating negative membrane curvature *in vitro*. However, the mechanisms by which MTSS1 localizes to intercellular junctions in epithelial cells and contributes to their integrity and maintenance have remained elusive. By carrying out EM and live-cell imaging on cultured Madin-Darby canine kidney cell monolayers, we provide evidence that adherens junctions of epithelial cells harbor lamellipodia-like, dynamic actin-driven membrane folds, which exhibit high negative membrane curvature at their distal edges. BioID proteomics and imaging experiments demonstrated that MTSS1 associates with an Arp2/3 complex activator, the WAVE-2 complex, in dynamic actin-rich protrusions at cell–cell junctions. Inhibition of Arp2/3 or WAVE-2 suppressed actin filament assembly at adherens junctions, decreased the dynamics of junctional membrane protrusions, and led to defects in epithelial integrity. Together, these results support a model in which membrane-associated MTSS1, together with the WAVE-2 and Arp2/3 complexes, promotes the formation of dynamic lamellipodia-like actin protrusions that contribute to the integrity of cell–cell junctions in epithelial monolayers.

The actin cytoskeleton plays important roles in plasma membrane dynamics. The coordinated polymerization of actin filaments provides the force for the generation of plasma membrane protrusions for cell migration and morphogenesis, as well as plasma membrane invaginations for endocytic processes (1, 2). During cell migration and morphogenesis, actin filaments can be arranged in parallel bundles or branched networks. The parallel filament bundles are typically nucleated and polymerized by formin and Ena/VASP family proteins, and they are critical for the formation of thin, needle-like filopodia and microspikes at the leading edge of motile cells (3). On the other hand, the branched actin filament networks are nucleated by the Arp2/3 complex and typically generate flat, lamellipodial plasma membrane protrusions at the leading edge of the cell (4, 5).

An important family of proteins regulating the interplay between the plasma membrane and the actin cytoskeleton in these processes are the Bin/amphiphysin/Rvs (BAR) domain proteins. These proteins are characterized by their lipid-binding, α-helical, dimeric BAR domain, which can sense or generate specific membrane curvatures. Additionally, BAR domain proteins are composed of other domains that link them to the actin cytoskeleton and various signaling pathways (6). BAR domain proteins can be further divided into three subgroups: canonical BAR domains, Fes/Cip4 homology BAR (F-BAR) domains, and inverse BAR (I-BAR) domains. These can sense and generate either positive membrane curvature (canonical BAR domains and F-BAR domains) to facilitate membrane invaginations, or negative curvature (I-BAR domains and a subset of F-BAR domains) to promote plasma membrane protrusions (7). Additionally, at least one I-BAR domain and one F-BAR domain protein prefer flat membrane geometry (8, 9).

Metastasis-suppressor 1 (MTSS1; also known as missing-in-metastasis) is an I-BAR domain protein, which senses and generates negative membrane curvature through its N-terminal I-BAR domain. MTSS1 additionally interacts with monomeric actin through its C-terminal Wiskott-Aldrich Syndrome homology region 2 (WH2) domain (10, 11). MTSS1 was originally identified as a tumor suppressor in bladder carcinoma (12). Subsequent studies have linked altered MTSS1 expression levels to the progression of a wide variety of cancers (13, 14, 15, 16). Although *Mtss1*-KO mice are viable, they are characterized by defects in neuronal and B-cell signaling (17, 18, 19, 20, 21). Moreover, MTSS1 is important for the formation of Arp2/3-dependent lamellipodia-like actin protrusions in endothelial cells that are critical for sealing transendothelial cell tunnels induced by bacterial toxins (22).

*Mtss1*-KO mice also suffer from progressive kidney defects caused by the compromised integrity of kidney epithelial intercellular junctions (23). Moreover, the depletion of MTSS1 from cultured epithelial cells leads to defects in the maintenance of cell–cell junctions (24). MTSS1 localizes to cell–cell junctions in cultured epithelial cells, and its overexpression induces actin filament accumulation at intercellular junctions of cultured epithelial cells, whereas *MTSS1* knockdown reduces the recruitment of actin filaments to cell–cell junctions (23, 24). However, the precise molecular function of MTSS1 in epithelial cell–cell junctions has been unknown. For example, the mechanism by which MTSS1 promotes actin filament assembly at intercellular junctions has remained elusive. Moreover, because epithelial cell–cell junctions have been considered to harbor flat membranes sealing the two adjacent cells to each other, it is unclear how a protein sensing/generating a negative membrane curvature would localize to cell–cell junctions and facilitate their assembly or maintenance. Interestingly, two recent studies have provided evidence that epithelial adherens junctions are not entirely flat but appear to contain interdigitated actin-rich microspikes that contribute to cell–cell adhesion (25, 26).

Here, by carrying out electron tomography analysis on Madin-Darby canine kidney (MDCK) cell monolayers, we provide evidence that adherens junctions of epithelial cells contain lamellipodia-like plasma membrane protrusions. We further show that MTSS1 localizes to the membrane of actin-rich, dynamic protrusions in adherens junctions of epithelial cells and that these structures additionally harbor Arp2/3 complex, its activator WAVE-2 complex, and other regulators of actin dynamics. By using an Arp2/3 inhibitor and *Wasf2* (gene that encodes WAVE-2) KO cells, we provide evidence that the WAVE-2:Arp2/3-dependent actin polymerization is important for these dynamic actin-based protrusions and for maintaining the integrity of intercellular junctions in epithelial monolayers. These results elucidate the mechanism by which MTSS1 localizes to cell–cell junctions and contributes to the maintenance of epithelial intercellular junctions by promoting actin filament assembly in association with WAVE-2 and Arp2/3 complexes.

## Results

### MTSS1 localizes to actin-rich structures at adherens junctions in polarized epithelial cells

**Figure 1:**
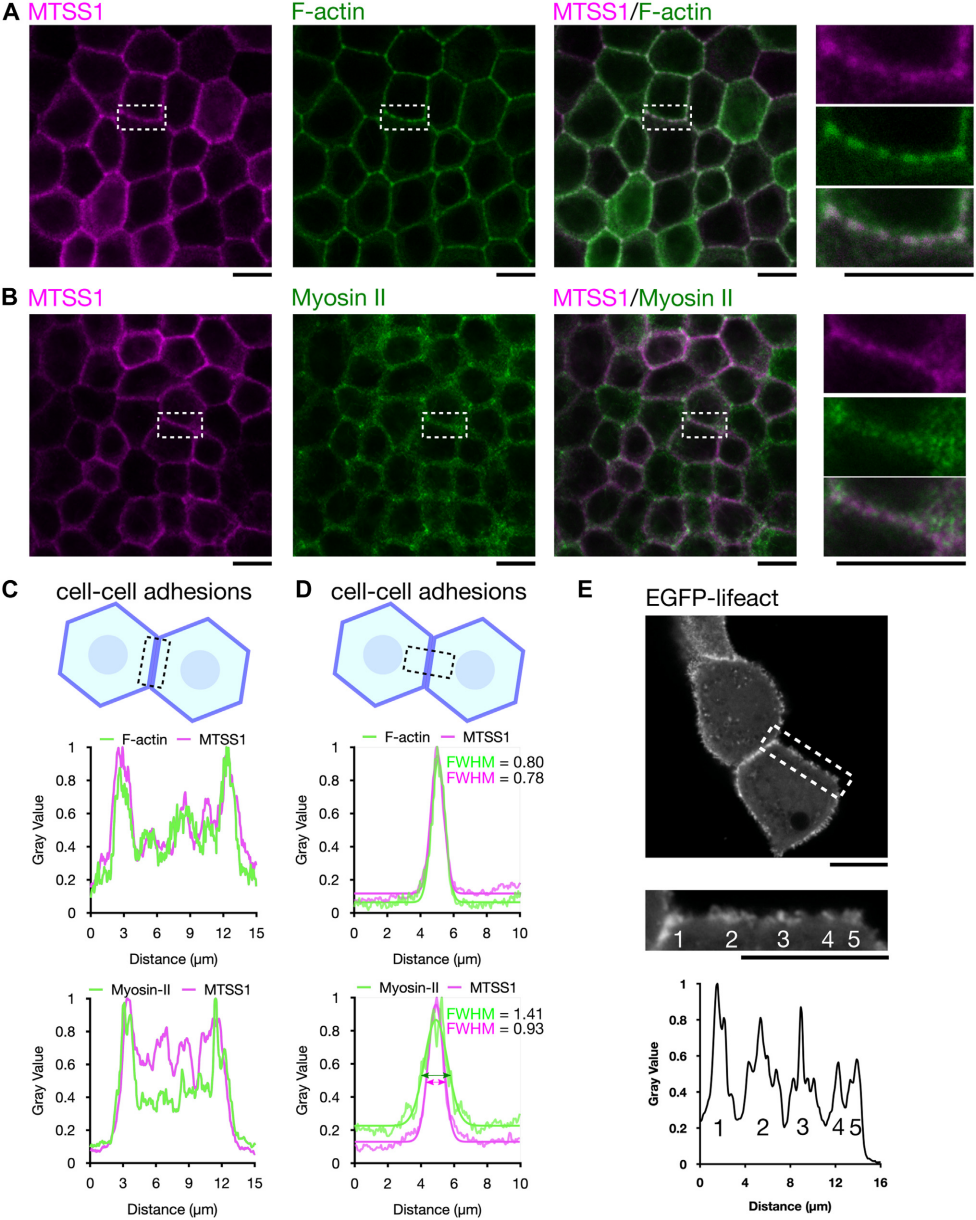
**The I-BAR domain protein MTSS1 localizes to actin-rich foci at intercellular junctions.***A*, colocalization of MTSS1 (*magenta*) with Alexa Fluor 488 phalloidin-labeled F-actin (*green*) in polarized MDCK cells stably expressing HA-tagged MTSS1 (*left*), observed using confocal microscopy. The boxed regions are enlarged and shown (*right*). MTSS1 coclusters with F-actin (indicated by *white*) at adherens junctions (The scale bar represents 10 μm). *B*, immunofluorescence staining of the colocalization of MTSS1 (*magenta*) with nonmuscle myosin IIA (*green*) in polarized MDCK cells stably expressing HA-tagged MTSS1 (*left*), observed using confocal microscopy. The boxed regions are enlarged and are shown (*right*). Myosin II localizes further away from the intercellular junctions compared to MTSS1 at adherens junctions (The scale bar represents 10 μm). *C*, normalized fluorescence intensities along the boxed region in the model diagram (*top*) indicate that MTSS1 (*middle*) but not myosin II (*bottom*) coclusters with F-actin at adherens junctions, as shown by peaks overlapping at the same locations along the line profiles. *D*, normalized fluorescence intensities along the boxed region in the model diagram (*top*) indicate that myosin II (*bottom*, FWHM = 1.41) localizes further away from the intercellular junctions compared to MTSS1 (*middle*, FWHM = 0.93) at adherens junctions. The line profiles were fitted with Gaussian curves, and FWHM (Full Width at Half Maximum, μm) was estimated. *E*, MDCK cells expressing EGFP-tagged LifeAct and generating actin protrusions in a confluent monolayer. The boxed region is enlarged and displayed at *below*. The normalized fluorescence intensity of the line profile along adherens junctions indicates that membrane protrusions are generated from the F-actin clusters. Each number from one to five in the line profile corresponds to the number of each F-actin cluster at cell–cell junctions (The scale bar represents 10 μm). I-BAR, inverse BAR; MDCK, Madin-Darby canine kidney; MTSS1, Metastasis-suppressor 1.

**Figure 2:**
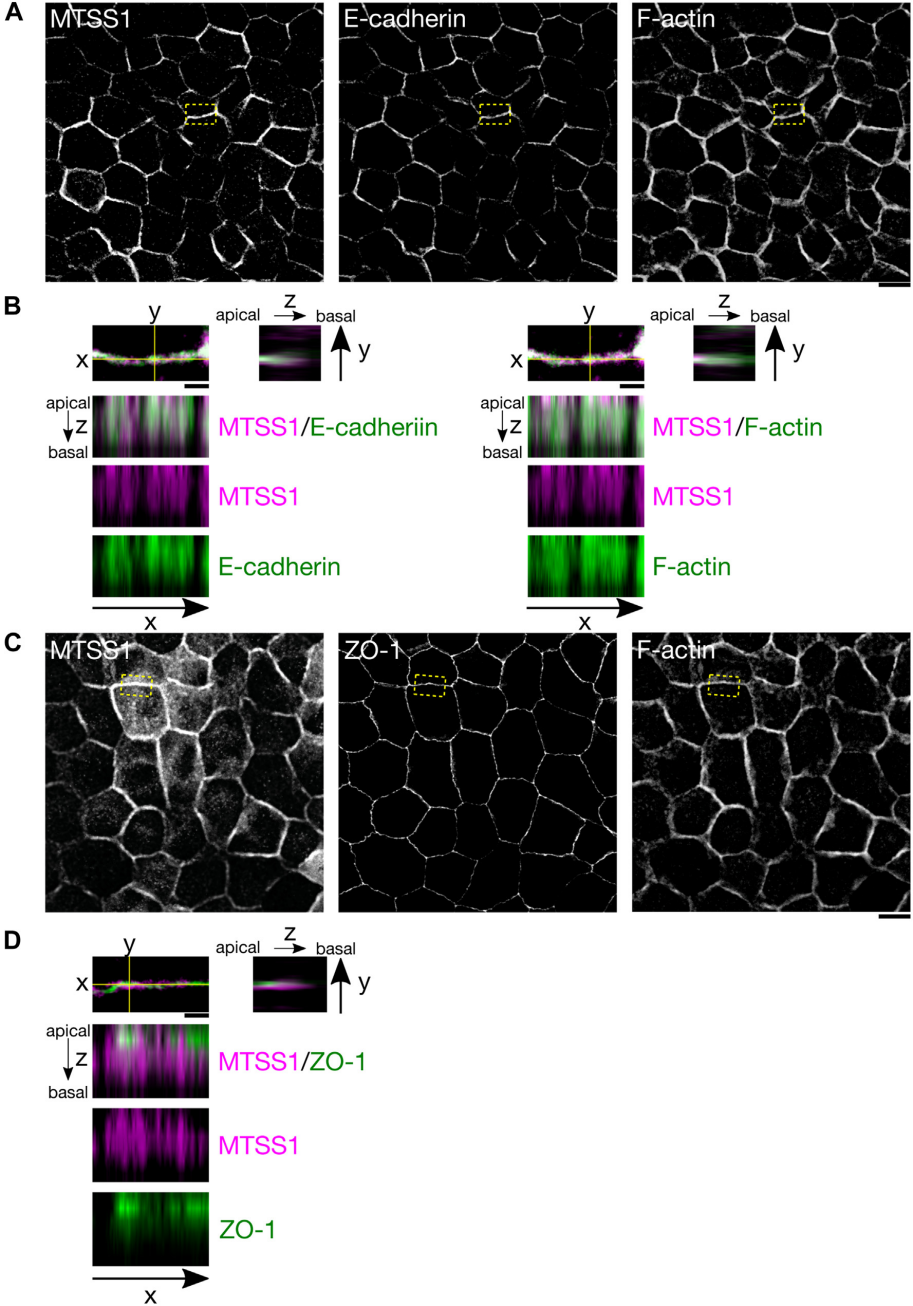
**MTSS1, F-actin, and E-cadherin colocalize at adherens junctions of epithelial cells.***A*, immunofluorescence images of MDCK cell monolayers stably expressing HA-tagged MTSS1. Adherens junctions were visualized with anti-E-cadherin antibody and F-actin with fluorescent phalloidin (The scale bar represents 10 μm). *B*, magnifications (from the regions indicated by *yellow boxes* in panel *A*) shown as composites and as orthogonal y-z and x-z projections along the *yellow lines* in the *upper panel* (The scale bar represents 2 μm). *C*, immunofluorescence images of MDCK cells stably expressing HA-tagged MTSS1. Tight junctions were visualized with anti-ZO-1 antibody and F-actin with fluorescent phalloidin (The scale bar represents 10 μm). *D*, magnifications (from the regions indicated by *yellow boxes* in panel *C*) shown as composites and orthogonal y-z and x-z projections along the *yellow lines* in the *upper panel* (The scale bars represent 2 μm). MDCK, Madin-Darby canine kidney; MTSS1, Metastasis-suppressor 1.

A previous study provided evidence that MTSS1 localizes predominantly to adherens junctions in epithelial cells (23), whereas another I-BAR domain protein IRSp53 accumulates in epithelial apical tight junctions (27, 28). To elucidate the molecular mechanisms by which MTSS1 localizes to adherens junctions, we established a stable MDCK cell line expressing an HA-tagged MTSS1. This was necessary because none of the available commercial antibodies that we tested specifically recognized the MTSS1 protein in epithelial cells so far. Confocal immunofluorescence microscopy revealed that HA-tagged MTSS1 colocalized with F-actin and E-cadherin at intercellular junctions, whereas MTSS1 and myosin II signals only partially overlapped at intercellular junctions (Figs. 1, *A*–*D* and S1, *A*–*C*). MTSS1 displayed similar vertical localization at cell–cell junctions as the adherens junction protein E-cadherin, but MTSS1 accumulated predominantly below the tight junction protein ZO-1 at intercellular junctions, demonstrating that it indeed localizes to adherens junctions in epithelial cells (Fig. 2, *A*–*D*). Importantly, both MTSS1 and F-actin were present as small, overlapping foci at intercellular junctions, indicating that MTSS1 associates with actin filament arrays at adherens junctions (Figs. 1*A*). On the other hand, the different localization patterns of myosin II and MTSS1 at adherens junctions suggest that myosin II–containing junctional actin filament bundles are distinct from MTSS1-containing actin filament structures at adherens junctions (Fig. S2, *A* and *B*). In support of this, 3D Structured illumination super-resolution microscopy experiments provided evidence that two types of actin-based structures exist at adherens junctions: actin-rich puncta and stress fiber-like actin filament bundles (Fig. S2*C*). Thus, we further investigated the junctional actin-rich puncta using live imaging of MDCK cells expressing EGFP-tagged LifeAct. These experiments provided evidence that the F-actin clusters at intercellular junctions may be associated with the formation of membrane protrusions (Fig. 1*E*). Finally, the PLC delta-PH domain that binds PI(4,5)P_2_, which is one of the phosphoinositides that enhances the interaction of the MTSS1 I-BAR domain with membranes, also localized as clusters at cell–cell junctions (Fig. S2*D*). Thus, PI(4,5)P_2_ may generate microdomains at cell–cell junctions and recruit I-BAR domain proteins through electrostatic interactions. Together, these data confirm the earlier observations on the localization of MTSS1 to adherens junctions (23) and further demonstrate that MTSS1 accumulates at actin-rich foci in intercellular junctions, which are distinct from junctional actomyosin bundles.

### Adherens junctions contain highly curved lamellipodia-like membrane structures

**Figure 3:**
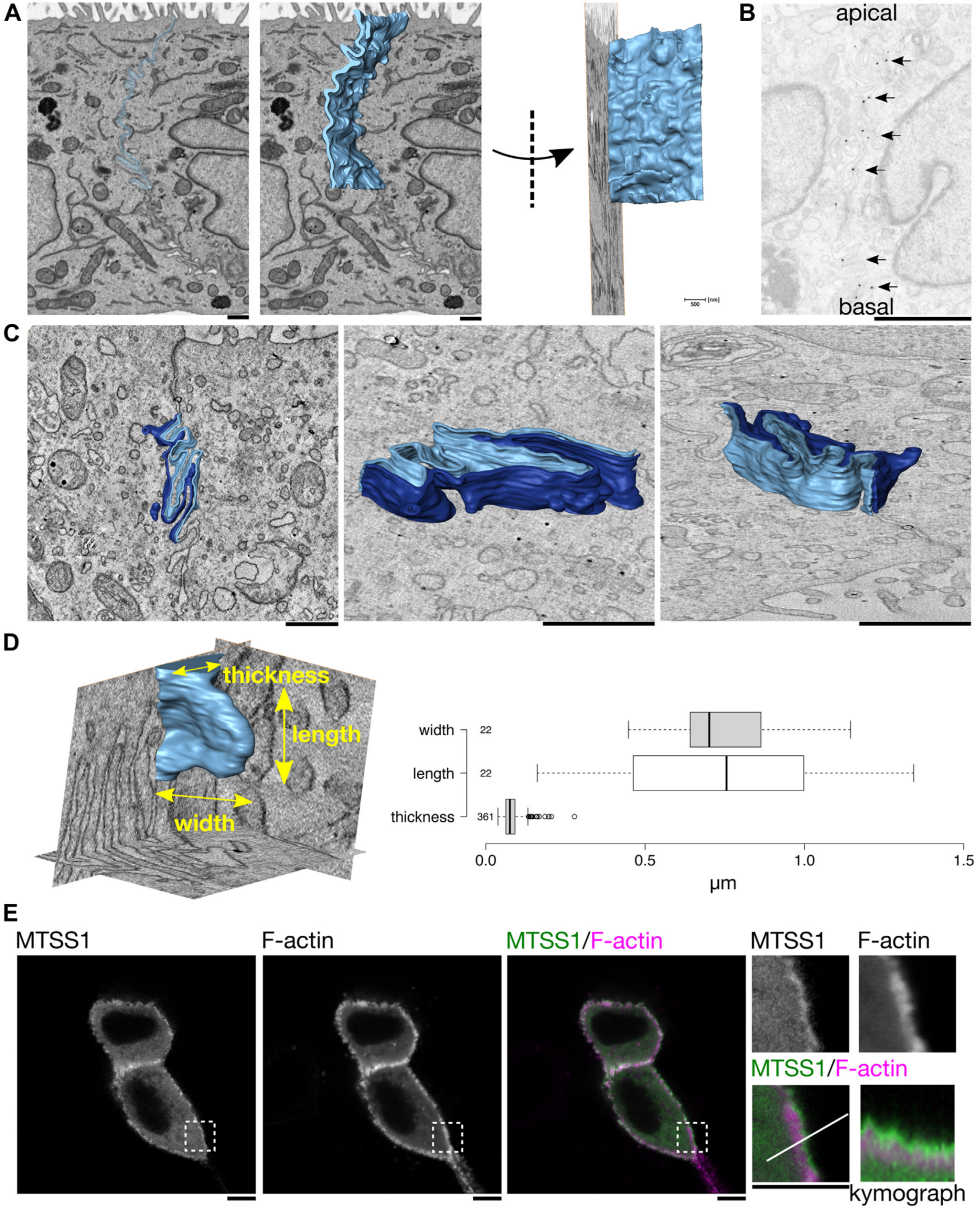
**Adherens junctions contain highly curved lamellipodia-like membrane protrusions.***A*, the highly curved membrane structure (*light blue*) at cell–cell junctions in polarized MDCK cells imaged by serial block-face scanning electron microscopy (SBEM, *left*). The modeled membrane shown in the *left image* is rotated approximately 90 degrees (*right*) (The scale bar represents 500 nm). *B*, the immunoelectron microscopy image of polarized MDCK cells stably expressing HA-tagged MTSS1. Sections were labeled with the HA antibody against MTSS1 and then labeled with gold particles indicated with *arrows* (The scale bar represents 2 μm). *C*, the 3D reconstruction of membrane structures at cell–cell junctions in polarized MDCK cells, indicated in *light* and *dark blue*, respectively, by electron tomography (*left*). Neighboring membranes are enlarged and viewed from the side to show the lamellipodia-like highly curved membrane structures (*middle* and *right*) (The scale bar represents 500 nm). *D*, the width, length, and thickness of the lamellipodia-like membrane protrusions at cell–cell junctions are defined in the electron tomographic model (*yellow*) and quantified in the images taken by SBEM. *Center lines* show medians; *box* limits indicate 25th and 75th percentiles as determined by R software; *whiskers* extend 1.5 times the interquartile range from the 25th and 75th percentiles (52). n = 22 (width), 22 (length), and 361 (thickness). *E*, live imaging of MDCK cells coexpressing EGFP-tagged full-length MTSS1 (*green*) and mCherry-tagged LifeAct (*magenta*) in a confluent monolayer, observed by confocal microscopy. The boxed regions are enlarged and shown (*right*). A kymograph was generated along the line profile (length = 4 min 49.795 s). Note that MTSS1 localizes closer to the leading edge of the cells than F-actin in lamellipodia-like membrane protrusions at cell–cell junctions (The scale bar represents 5 μm). MDCK, Madin-Darby canine kidney; MTSS1, Metastasis-suppressor 1.

MTSS1 and other I-BAR domain proteins can generate and sense a negative membrane curvature (6, 29, 30, 31, 32), and in endothelial cells, MTSS1 localizes to lamellipodia-like actin-rich structures (22). Thus, we examined the ultrastructure of the membrane morphology at intercellular junctions of MDCK cells using transmission electron microscopy (TEM), electron tomography, and serial block face scanning electron microscopy (Fig. 3*A* and Movie S1–S4). As reported recently (25, 26), the morphology of the plasma membrane at adherens junctions in polarized epithelial cells is not flat but rather highly curved. Immuno-EM studies on cells stably expressing HA-tagged MTSS1 revealed that MTSS1 localizes to the vicinity of the curved plasma membrane at the adherens junctions of MDCK cells (Fig. 3*B*). Next, we characterized the morphology of the intercellular junctions in more detail using 3D-EM and found that these membranes are indeed highly curved (Fig. 3*C*). Importantly, our findings revealed that adherens junctions are enriched by lamellipodia-like membrane protrusions that have an average of approximately 80 nm thickness, 800 nm length, and 850 nm width (Fig. 3*D*). In contrast to earlier studies (25, 26), our 3D-EM analysis revealed lamellipodia-like membrane protrusions more frequently than filopodia/microspike-like thin protrusions at adherens junctions.

To study the possible interplay between MTSS1 and actin at protrusive structures of adherens junctions, we performed live imaging of MDCK cells coexpressing EGFP-tagged MTSS1 and mCherry-tagged LifeAct (Fig. 3*E* and Movie S5–S7). These studies further confirmed the presence of dynamic actin-rich protrusions at intercellular junctions. Interestingly, these experiments revealed that MTSS1 was enriched closer to the leading edge of the protrusions than actin, suggesting that MTSS1 localizes to the plasma membrane in the front of the actin filament network. MTSS1 may thus promote actin filament assembly or link the actin filament network to the plasma membrane. Together, these results demonstrate that epithelial adherens junctions contain lamellipodia-like membrane protrusions and suggest that these curved membrane regions correspond to dynamic MTSS1- and actin-rich protrusions.

### BioID-analysis of MTSS1 interactome in epithelial cells

**Figure 4:**
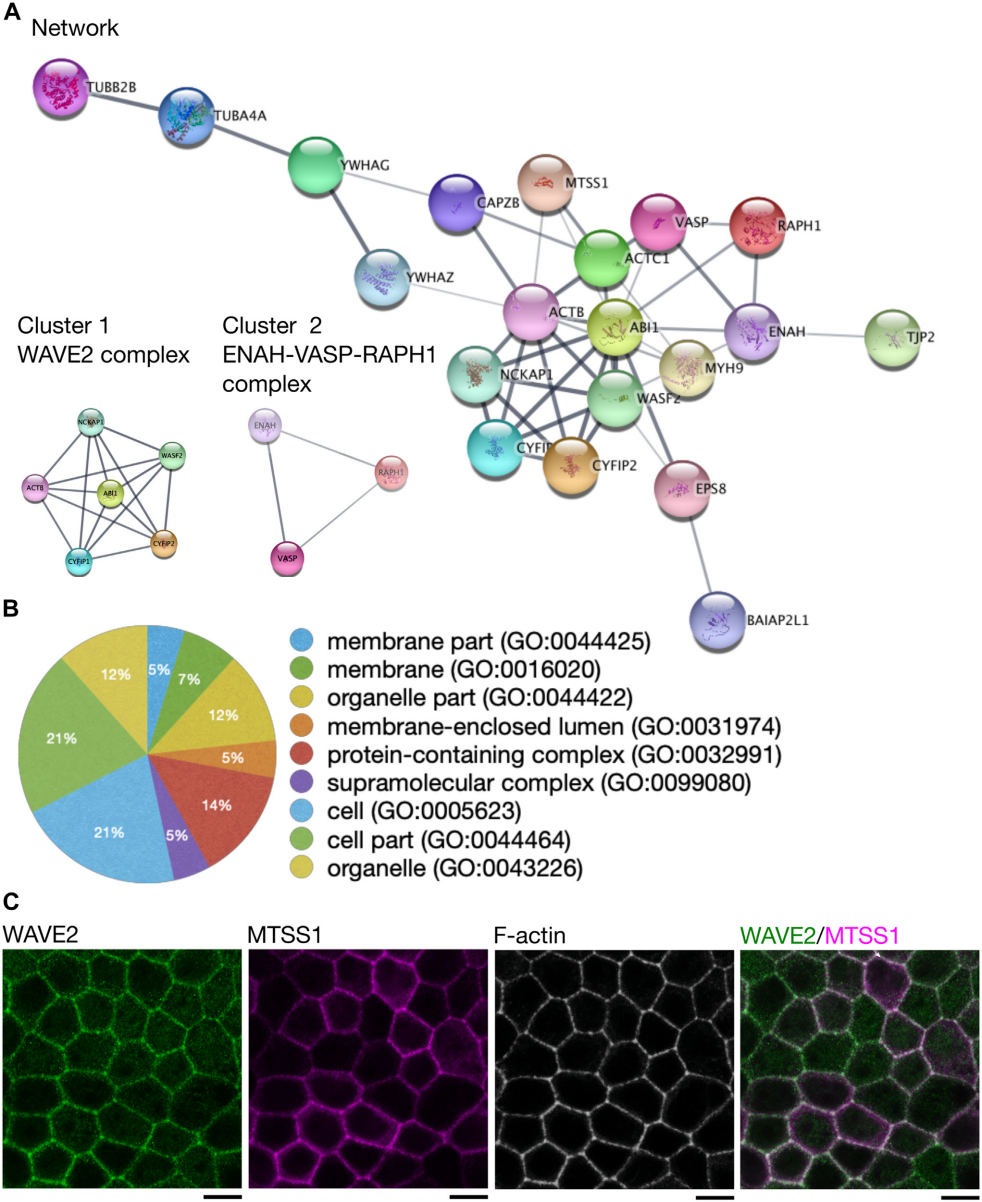
**Identification of MTSS1 interaction partners at adherens junctions.***A*, MTSS1 interaction partners at adherens junctions in polarized MDCK cells stably expressing BirA-tagged MTSS1 were identified using proteomics approach, BioID. Protein-protein interaction network analysis using stringApp in Cytoscape was performed in the result obtained from the BioID. Protein complexes (*i.e.*, WAVE-2 and ENAH-VASP-RAPH1 complexes) in the protein-protein interaction network were identified using MCODE in Cytoscape. *B*, functional analysis of the identified protein–protein interactions by PANTHER indicates that a large number of proteins identified as the putative interaction partners of MTSS1 function close to the membrane by forming protein complexes. *C*, immunofluorescence staining of the colocalization of WAVE-2 (*green*) with MTSS1 (*magenta*) and Alexa Fluor 488 phalloidin-labeled F-actin (*gray*) in polarized MDCK cells (The scale bar represents 10 μm), observed using confocal microscopy. Note that MTSS1, WAVE-2, and F-actin cocluster at adherens junctions. MDCK, Madin-Darby canine kidney; MTSS1, Metastasis-suppressor 1.

To elucidate the interplay between membrane-binding MTSS1 and the actin cytoskeleton in epithelial cells, we studied the interactome of MTSS1 in MDCK cells using the proximity-dependent biotin (BioID) approach (33). A construct encoding biotin ligase fused to the C-terminus of MTSS1 was expressed in MDCK cells, and biotinylated proteins were subsequently identified using a proteomics approach. For MTSS1, the proximal and interacting proteins were actin and several actin-regulatory proteins, including VASP, the beta subunit of heterodimeric capping protein, and several subunits of the WAVE-2 complex (Table 1). Protein-protein interaction network analysis further identified two specific clusters of actin regulators, namely the Arp2/3 activator WAVE-2 complex and the ENAH–VASP–RAPH1 complex (Fig. 4, *A* and *B*).

**Table 1:** List of the genes and proteins of putative interaction partners of MTSS1 identified by BioID.

Accession	PSMs	Gene	Protein
F1PKA2	108	*MTSS1*	MTSS I-BAR domain containing 1
F1PRR4	38	*VASP*	Vasodilator-stimulated phosphoprotein
O18840	35	*ACTB*	Actin, cytoplasmic 1
F1PDQ4	33	*CYFIP1*	Cytoplasmic FMR1-interacting protein
F1PH57	31	*NCKAP1*	NCK associated protein 1
P50551	31	*VASP*	Vasodilator-stimulated phosphoprotein
F1PQ68	30	*TUBB2B*	Tubulin beta chain
F1PWE6	26	*BAIAP2L1*	BAI1 associated protein 2 like 1
F1PMM7	25	*CYFIP2*	Cytoplasmic FMR1-interacting protein
F2Z4N7	20	*ACTC1*	Actin alpha cardiac muscle 1
E2RBC3	18	*TUBA4A*	Tubulin alpha chain
J9NUZ5	15	*RAPH1*	Ras-associated and pleckstrin homology domains-containing protein 1
J9P1R0	12	*EPS8*	Epidermal growth factor receptor pathway substrate 8
E2RR96	11	*WASF2*	WASP family member 2
F1Q0U7	11	*ENAH*	ENAH actin regulator
E2RMI1	10	*ABI1*	Abl interactor 1
F1PBL1	10	*YWHAZ*	14_3_3 domain-containing protein
F1PQS3	9	*CAPZB*	F-actin-capping protein subunit beta
F1P9J3	9	*MYH9*	Non-muscle myosin heavy chain IIa
J9NRH5	9	*YWHAG*	14-3-3 protein gamma-1
J9P749	8	*TJP2*	Tight junction protein ZO-2

Abbreviation: PSMs, peptide spectrum matches.

Confocal microscopy revealed that the endogenous WAVE-2 complex colocalized with MTSS1 and F-actin at adherens junctions of polarized epithelial cells and that these three proteins coclustered with each other at small foci (Figs. 4*C* and S3). Moreover, the other actin-regulatory complex, ENAH–VASP–RAPH1, colocalized with MTSS1 and F-actin at adherens junctions in polarized epithelial cells (Fig. S4).

### Arp2/3-mediated actin polymerization is important for maintaining the integrity of adherens junctions

Because previous studies have linked MTSS1 to Arp2/3-dependent actin polymerization in endothelial cells (22), and because Arp2/3 activator WAVE-2 complex components were among the top hits in the MTSS1 BioID screen, we further investigated the role of Arp2/3-dependent actin polymerization at epithelial cell–cell junctions. Polarized MDCK cells were treated with DMSO (control), an Arp2/3 specific inhibitor CK-666, or a myosin II specific inhibitor (−)-blebbistatin (Fig. S5, *A* and *B*). Inactivation of the Arp2/3 complex in MDCK cells diminished the F-actin intensity at adherens junctions, and the junctional F-actin clusters observed in the control cells disappeared. Inactivation of myosin II also diminished F-actin accumulation at adherens junctions; however, F-actin clusters at the cell–cell junctions were still present. These results provide further evidence that there are indeed two types of actin filament structures at the cell–cell junctions: Arp2/3-dependent actin filament foci at the plasma membrane and myosin II–dependent junctional actin filament bundles. The results obtained from CK-666–treated cells further suggest that Arp2/3 is largely responsible for the formation of F-actin clusters at the cell–cell junctions.

**Figure 5:**
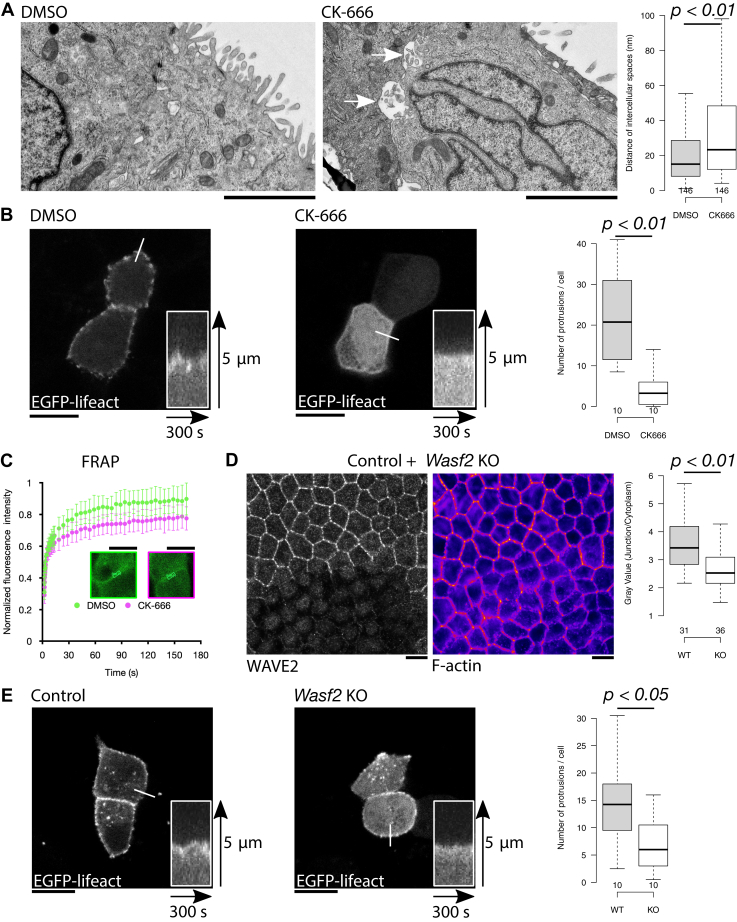
**The Arp2/3 complex is important for the integrity of adherens junctions.***A*, TEM micrographs of cell–cell junctions in polarized MDCK cells treated with DMSO (control) or CK-666 (Arp2/3 complex specific inhibitor). Note that the intercellular spaces (*arrows*) were observed in Arp2/3 complex–inactivated cells. Distances of the clearly identified intercellular spaces between adjacent MDCK cells treated with DMSO (control) or CK-666 were measured (*right*). *Center lines* show medians; *box* limits indicate the 25th and 75th percentiles as determined using R software; *whiskers* extend 1.5 times the interquartile range from the 25th and 75th percentiles. To facilitate comparison with the DMSO control, the Y-axis range was adjusted to display the interquartile range (IQR) clearly. n = 146 (DMSO) and 146 (CK-666) (The scale bar represents 2 μm). *B*, kymographs were generated along line profiles in DMSO- (control) or CK-666–treated MDCK cells expressing EGFP-tagged LifeAct (a marker to visualize F-actin) in a confluent monolayer, observed by time-lapse imaging using confocal microscopy. Control cells produce dynamic membrane protrusions, whereas Arp2/3 complex–inactivated cells have less membrane protrusions (The scale bar represents 10 μm). The number of membrane protrusions at cell–cell junctions was quantified in DMSO- (control) and CK-666–treated MDCK cells (right). n = 10 (DMSO) and 10 (CK-666) measurements. *C*, MDCK cells expressing EGFP-tagged actin were treated with DMSO (control) or CK-666, and actin dynamics were analyzed by fluorescence recovery after photobleaching (FRAP). The *boxed regions* (ROI) were photobleached, and fluorescence recovery was measured by confocal microscopy. n = 15 (DMSO) and 17 (CK-666). Note that the actin turnover in cell–cell junctions was suppressed in Arp2/3 complex–inactivated cells. (The scale bar represents 10 μm). *D*, F-actin intensities in CRISPR/Cas9 control and *Wasf2* (gene that encodes WAVE-2) KO polarized MDCK cells mixed with each other. The *left panel* demonstrates the identification of *Wasf2* KO cells by immunofluorescence staining with a WAVE-2 specific antibody, and the *right panel* shows the intensity of Alexa Fluor 488 phalloidin-labeled F-actin in these cells. Ratios (junction/cytoplasm) of F-actin fluorescence intensities at adherens junctions in CRISPR/Cas9 control, and *Wasf2* KO–polarized MDCK cells indicate decreased F-actin assembly at adherens junctions when WAVE-2 is absent. n = 31 (WT) and 36 (KO). (The scale bar represents 10 μm). *E*, live imaging of F-actin at cell–cell junctions in EGFP-tagged LifeAct-expressing MDCK cells in a confluent monolayer in which *Wasf2* was knocked out by CRISPR-Cas9, observed using confocal microscopy. The kymograph was generated along the line profile. Note that fewer actin protrusions were observed at cell–cell junctions in WAVE2-inactivated cells (The scale bar represents 10 μm). Frequencies of actin protrusions at cell–cell junctions were quantified in CRISPR/Cas9 control or *Wasf2*-KO MDCK cells. n = 10 (Control) and 10 (*Wasf2* KO). MDCK, Madin-Darby canine kidney; TEM, transmission electron microscopy.

Next, we used TEM to investigate the membrane ultrastructure at cell–cell junctions of polarized epithelial cells in which the Arp2/3 complex was inactivated (Fig. 5*A*). The cell–cell junctions were impaired, with frequent spaces between neighboring cells in the Arp2/3-inactivated cells. These results suggest that Arp2/3-mediated actin filament assembly is important for sealing and maintaining the integrity of epithelial cell–cell junctions.

### Dynamic actin-rich protrusions at intercellular junctions are dependent on Arp2/3 and WAVE-2 complexes

As Arp2/3-mediated actin filament assembly is important for the integrity of cell–cell junctions, we next investigated the dynamics of junctional actin-rich protrusions by time-lapse microscopy. Thus, we performed live imaging of MDCK cells expressing EGFP-tagged LifeAct in the presence of DMSO (control, Movie S8) or the Arp2/3 specific inhibitor CK-666 (Fig. 5*B* and Movie S9). Whereas control cells exhibited frequent, dynamic actin-rich protrusions at intercellular junctions, the Arp2/3-inactivated cells were unable to generate prominent membrane protrusions, as previously reported for actin-rich microspikes in epithelial cells (25). Importantly, the lamellipodia-like membrane protrusions observed in control cells occurred from the F-actin clusters at adherens junctions (Fig. 1*E*), which also contain MTSS1 and the WAVE-2 complex based on our experiments (Fig. 4*C*). Further, we investigated actin dynamics within these structures using fluorescence recovery after photobleaching (Fig. 5*C*) and found that actin filament turnover was diminished in Arp2/3-inactivated epithelial cells, although it was not completely halted. Thus, F-actin clusters at cell–cell junctions are Arp2/3-dependent, and actin turnover in these clusters is dynamic (Fig. S5*C*). This further indicates the presence of at least two different actin filament populations generated by different assembly mechanisms at epithelial cell–cell junctions.

Since proteomic analysis revealed that the Arp2/3 activator WAVE-2 is a putative interaction partner of MTSS1, we generated *Wasf2* (gene that encodes WAVE-2) KO MDCK cell lines using the CRISPR-Cas9 approach (Fig. S6) and investigated the organization and dynamics of actin filaments at adherens junctions of polarized *Wasf2* KO cells. By mixing control cells and *Wasf2* KO cells with each other, we learned that in fully confluent epithelial monolayers, the amount of filamentous actin at adherens junctions was diminished in WAVE-2 depleted cells (Figs. 5*D* and S7). Time-lapse imaging of EGFP-LifeAct–expressing *Wasf2* KO cells also revealed that the dynamics of actin-rich protrusions were suppressed in the absence of WAVE-2 compared to those of CRISPR-Cas9 control MDCK cells (Fig. 5*E*). Together, these results demonstrate that the WAVE-2:Arp2/3-mediated actin filament assembly is critical for the formation of dynamic actin-rich protrusions at intercellular junctions, and this molecular complex contributes to the integrity of adherens junctions in epithelial cells.

## Discussion

The molecular mechanisms by which actin filament arrays, together with proteins that sense and generate membrane curvature, function at the intercellular junctions of epithelial cells have remained elusive. Here, we reveal that epithelial adherens junctions contain flat, lamellipodia-like membrane protrusions. We further showed that the membrane curvature-sensing/generating I-BAR domain protein (MTSS1) and the Arp2/3 activator (WAVE-2 complex) localize to dynamic, actin-rich protrusions at intercellular junctions and that both WAVE-2 and Arp2/3 complexes are critical for these actin-based protrusions. Combined with previous work on the role of MTSS1 in actin assembly and integrity of epithelial cell–cell junctions (23), these data support a model where MTSS1:WAVE-2:Arp2/3-dependent, membrane curvature–sensing actin polymerization machinery is important for the formation of lamellipodia-like membrane protrusions at intercellular junctions.

Although according to a popular view, membranes at the intercellular junctions of epithelial cells are relatively flat, recent thin-section EM studies provided evidence for the presence of thin, microspike-like protrusions at epithelial cell–cell junctions (25). They also demonstrated that the cadherin puncta at adherens junctions correspond to interdigitated actin microspikes that would increase the surface area at the junctions between epithelial cells (26). However, our 3D electron tomography analysis rather showed that cell–cell junctions contain flat, Arp2/3 complex–dependent lamellipodia-like membrane protrusions that result in a folded architecture of the cell–cell interface. The differences between our study and these two recent publications (25, 26) may have resulted from differences in the confluency of epithelial monolayers. Thus, microspikes may be more frequent during monolayer formation and repair, while lamellipodial protrusions could be more important for the mechanical reinforcement of epithelial integrity through the efficient enlargement of the contact area at the established intercellular junctions in fully confluent monolayers. Alternatively, these differences may have arisen from techniques used. While the cell–cell junctions in the previous study (25) were analyzed by 2D thin-section EM, we applied electron tomography and volume EM to determine the 3D architecture of membranes at cell–cell junctions. Thus, the microspike-like structures observed by 2D thin-section approach may actually be cross-sections of flat lamellipodia-like protrusions that were revealed by our 3D-EM analysis. It is also important to note that the lamellipodia-like protrusions identified by our 3D-EM analysis are distinct from the recently discovered “cryptic lamellipodia” that are only present at the basal cell–matrix interfaces of epithelial cells and contribute to the migration of epithelial sheets (34).

The WAVE-2 and Arp2/3 complexes are important for actin assembly and junctional integrity in epithelial cells (35, 36, 37). Our study provides evidence that both Arp2/3 and WAVE-2 complexes localize to actin-rich junctional protrusions in epithelial cells and that they are important for the dynamics of these membrane protrusions. Moreover, recent studies have demonstrated that the Arp2/3 complex is important for the formation of interdigitated actin-rich microspikes during epithelial repair (25, 26). Importantly, the lamellipodia- and microspike-like protrusions at adherens junctions do not precisely colocalize with myosin II–dependent junctional actin filament bundles. These structures also respond differently to Arp2/3 inhibition and appear to have different functions in epithelial cells. Thus, our findings, together with the earlier study (25, 26), suggest that adherens junctions are composed of at least two different types of actin filament arrays, which are assembled through different mechanisms and functionally distinct. In this context, it is important to note that endothelial cell–cell junctions are composed of both linear actomyosin bundles and Arp2/3-dependent, branched actin filament arrays that push the plasma membrane (38).

The presence of lamellipodia-like protrusions, which exhibit highly curved edges, at adherens junctions provides a plausible explanation for how MTSS1 localizes to epithelial cell–cell junctions. We propose that MTSS1 senses negative membrane curvature at PI(4,5)P_2_-rich regions of plasma membrane through its I-BAR domain. This is expected to induce PI(4,5)P_2_-clustering (30) that may enhance the accumulation of more MTSS1 and other phosphoinositide-sensing regulators of actin filament assembly (39) in the region of the negative membrane curvature. MTSS1 may then either directly or indirectly recruit the WAVE-2 complex and Arp2/3 to the site of negative membrane curvature and hence trigger actin filament assembly. This would push the plasma membrane forward to generate a dynamic lamellipodia-like protrusion. Because the Arp2/3-dependent protrusions are important for the integrity of epithelial cell–cell junctions, this model provides a plausible explanation for the earlier observation on the role of MTSS1 in maintaining epithelial integrity (23). Moreover, the role of MTSS1 in plasma membrane protrusions, which stabilize epithelial junctions, suggests that in carcinoma cells expressing only low levels of MTSS1, the junctional integrity would be compromised. This could enhance the epithelial-to-mesenchymal transition and thus explain why low MTSS1 expression levels are linked to metastasis in multiple carcinomas (13, 14).

Collectively, our study proposes that MTSS1:WAVE-2:Arp2/3-dependent, lamellipodia-like actin-rich protrusions are important for the stability of epithelial cell–cell junctions. Combined with earlier studies, this study provides evidence that intercellular junctions of epithelial cells and leading edges of migrating mesenchymal cells share structurally similar actin filament arrays, namely flat membrane protrusions composed of a branched actin filament network, thin filopodia-like actin filament bundles, and contractile actomyosin bundles. In the future, it will be important to determine the precise mechanisms underlying the MTSS1–WAVE-2 interaction, *i.e.*, whether these two proteins interact directly with each other or whether this interaction is mediated by another protein. Moreover, because Ena/VASP family proteins localize to both lamellipodia and filopodia in mesenchymal cells (40, 41) and have been implicated in actin dynamics in epithelial cells (42), the precise role of Ena/VASP proteins and their interaction partners in epithelial cell–cell junctions remains to be determined.

## Experimental procedures

### Subcloning

Sequences encoding MTSS1 and PLC delta-PH were subcloned into the pEGFP-N1 vector (Clontech). The sequences encoding LifeAct and actin were subcloned into the pEGFP-C1 and pmCherry-C1 vectors (Clontech). The sequence encoding MTSS1 was subcloned into the pcDNA3.1(−) vector (Thermo Fisher Scientific) containing a C-terminal HA-tag sequence. The sequence encoding myc-BirA (control) or MTSS1 containing a C-terminal myc-BirA was subcloned into the pcDNA3.1(−) vector.

### Cell culture

MDCK II cells (European Collection of Authenticated Cell Cultures) were cultured in minimum essential medium (MEM) supplemented with 5% fetal bovine serum (Invitrogen), L-glutamine (Sigma-Aldrich), and penicillin/streptomycin (Sigma-Aldrich) according to the supplier’s suggested protocol. *Mycoplasma* contamination in cell cultures was routinely tested using the MycoAlert *Mycoplasma* Detection Kit (Lonza). For polarization, MDCK cells were cultured in Transwell polyester membrane cell culture inserts with a 0.4 μm pore polyester membrane (Corning) coated with collagen (Sigma-Aldrich) (43).

### Transfection

Transfections were performed with FuGENE HD (Promega) using Opti-MEM (Invitrogen) in the absence of antibiotics, according to the manufacturer’s protocol. For stable transfection, MDCK cells were transfected with an HA-tagged full-length MTSS1 plasmid containing the neomycin resistance gene (*neo*) for G418 (Geneticin) resistance. Then, 48 h posttransfection, transfected cells were maintained for 2 to 3 weeks in a selective medium containing G418, which was replaced every 3 to 4 days until distinct colonies appeared. Individual colonies were then trypsinized and transferred to multiwell plates for limited dilution cloning in the presence of a selective medium. Isolated individual clones containing the transfected DNA were verified by Western blotting, and the clone with relatively low expression of HA-tagged MTSS1 was selected to avoid the gain-of-function phenotype due to the overexpression.

### Western blotting

Rabbit anti-WAVE-2 (D2C8) primary antibody (1:1000; Cell Signaling Technology, Inc, #3659), mouse anti-tubulin primary antibody (1:4000; Sigma-Aldrich, T5168), and mouse/rabbit anti-HRP secondary antibodies (1:10,000; Thermo Fisher Scientific) were used for Western blotting, which was performed according to the standard protocol.

### CRISPR-Cas9 to generate gene KO cell lines

CRISPR was used to generate *Wasf2* KO cells by coexpressing an endonuclease, Cas9, and a guide RNA specific to the targeted gene with a unique ∼20 nucleotide DNA sequence compared to the rest of the genome present adjacent to a protospacer adjacent motif. Gene editing with Cas9 and single guide RNA–expressing lentivirus (LentiCRISPR) was performed as previously described (44, 45). Two separate target sequences from constitutive early exons were selected for each gene. Target sequences with no off-target sites with less than three mismatches in the *Canis lupus familiaris* genome were selected based on the FASTA - Sequence Similarity Searching (EMBL-EBI). The gRNA oligos with BsmBI (New England Biolabs) overhangs were subcloned into lentiCRISPR v2. To produce lentiviruses, 70 to 80% confluent 293T-D10 on CellBIND 10 cm Ø tissue culture dishes (Corning) were cotransfected with lentiCRISPR, pPAX2, and VSV-G plasmids using Lipofectamine 2000 reagent (Thermo Fisher Scientific) in Opti-MEM (Thermo Fisher Scientific). For infection, viral supernatants were used directly as described previously (44, 45). Subconfluent MDCK cells, seeded at a density of 6 × 10^4^/well of 24-well plate the previous day, were infected for 24 h, expanded for another 24 h without virus, and then trypsinized, reseeded, and cultured for 24 h in the presence of 6 μg/ml puromycin to select transduced cells. Clonal cell lines were established from the puromycin-resistant population. After the selection of candidate clones by limiting dilution cloning, the disruption of the target genes in the isolated clones was confirmed by sequencing the target regions, and the expression of target proteins in the obtained clones was examined by Western blotting and DNA sequencing.

Genomic DNAs from WT and *Wasf2* KO cells were extracted using a PureLink Genomic DNA Mini Kit (Invitrogen, K182002). WT and *Wasf2* KO single clones in exon 3 and exon 4 were sequenced with *Wasf2*-specific primers 5ʹ-TCAGCCTGCATTGAGTTCGT-3ʹ, 5ʹ-GCCTTGCTAGAATGTTGCCC-3ʹ, 5ʹ-CAGCCTGCATTGAGTTCGTG-3ʹ, and 5ʹ-CACAGGTTGACAAAGGGAGGA-3ʹ at Eurofins Genomics sequencing service (for Sanger sequencing). The clone where exon 3 was targeted was further analyzed by next-generation sequencing (NGS MiSeq) at the Institute of Biotechnology Sequencing unit at University of Helsinki using the primers 5ʹ-TGCACTAGTGACTTGGTTTCTTTG-3ʹ and 5ʹ-TTTCAAAGCAGAACTCCATAGGT-3ʹ (for NGS). Sequence analysis was performed with Geneious Prime 11.0.12 (Biomatters, Ltd).

### Immunofluorescence microscopy

The following antibodies were used for the immunofluorescence staining with the indicated dilutions: rabbit anti-HA antibody (1:200; Merck, H6908), mouse anti-HA antibody (1:500; Merck, H3663), rabbit anti-nonmuscle myosin heavy chain II-A antibody (1:1000; BioLegend, 909801), mouse anti-E-cadherin antibody (1:50; Cell Signaling Technology, Inc, #14472), rabbit anti-E-cadherin (24E10) antibody (1:50; Cell Signaling Technology, Inc, #3195S), rabbit anti-ZO-1 antibody (1:150; Cell Signaling Technology, Inc, #8193S), mouse anti-beta-Catenin antibody (1:300; Invitrogen, Inc #13-8400), rabbit anti-VASP antibody (1:400; Sigma-Aldrich, HPA005724), rabbit anti-RAPH1 antibody (1:200; Sigma-Aldrich, HPA020027), rabbit anti-ENAH antibody (1:400; Sigma-Aldrich, HPA028696), rabbit anti-WAVE-2 antibody (1:50; Cell Signaling Technology, Inc, #3659), Alexa 488 goat anti-rabbit IgG (heavy and light chains) highly cross-adsorbed secondary antibody (1:1000; Thermo Fisher Scientific), and Alexa Fluor 488/568/647 phalloidin (Thermo Fisher Scientific). Images were acquired using a TCS SP8 confocal microscope (Leica) with HC PL APO 63×/1.20 W motCORR CS2 and HC PL APO 93×/1.30 GLYC CORR STED WHITE objective. Line profiles were generated using the Fiji/ImageJ software (https://fiji.sc/) (NIH). All x-y images presented in the figures were obtained at approximately central vertical plane of cell–cell junctions.

### Live-cell imaging

Cells were cultured on glass-bottom dishes (Greiner Bio-One) coated with collagen (Sigma-Aldrich), and imaged using a 488-nm laser for EGFP and a 552-nm laser for mCherry with a TCS SP8 confocal microscope (Leica) with a Leica objective (HC PL APO 63×/1.20 W motCORR CS2) at 37 °C and under 5% CO_2_. Kymographs were generated using the Fiji/ImageJ software (NIH). CK-666 (Sigma-Aldrich) and (−)-blebbistatin (Sigma-Aldrich) were used at concentrations of 100 μM and 15 μM, respectively.

### Fluorescence recovery after photobleaching

Cells were cultured on glass-bottom dishes (Greiner Bio-One) coated with collagen (Sigma-Aldrich). Fluorescence recovery after photobleaching experiments were performed in a region of interest using a 488-nm laser with a TCS SP8 confocal microscope (Leica) with a Leica objective (HC PL APO 63×/1.20 W motCORR CS2) at 37 °C under 5% CO_2_. The time course of changes in the fluorescence intensity in a region of interest after photobleaching was followed by using excitation light at the minimum intensity to minimize the subsequent photobleaching. The intensities were normalized to the intensities before bleaching.

### 3D structured illumination microscopy

F-actin in MDCK cells was stained with Alexa Fluor 488 phalloidin (Thermo Fisher Scientific) according to the manufacturer’s protocol, mounted with VECTASHIELD Mounting Medium (Vector Laboratories), and observed using a structured illumination microscopy on an N-SIM equipped with an Apo TIRF objective, NA 1.49 (Nikon GmbH) using the 3D Structured illumination mode. Acquisition and reconstruction were performed with Nis-Elements 4.2 software (https://www.microscope.healthcare.nikon.com/products/software/nis-elements).

### Transmission EM, electron tomography, and volume EM

MDCK cells were cultured in Transwell cell culture inserts with a 0.4 μm pore polyester membrane (Corning) coated with collagen (Sigma-Aldrich) for polarization. Cells were then fixed with 2% glutaraldehyde (EM grade) in 0.1 M Na-cacodylate buffer (pH 7.4) supplemented with 2 mM CaCl_2_. After 30 min fixation, cells were washed and osmicated with 1% reduced osmium prior to dehydration and flat embedding into epoxy resin (TAAB). Resin was polymerized at 60 °C overnight, and a pyramid was trimmed in the direction enabling cell cross-sections. The 60 to 70 nm sections were picked up on Pioloform-coated, single slot grids, and poststained with uranyl acetate and lead citrate prior to imaging with a JEM 1400 microscope (JEOL) equipped with an Orius SC 1000B CCD camera (Gatan, Inc).

For electron tomography, specimens were prepared as described previously (46, 47). Dual axis tilt series, ±62 degrees at one-degree intervals, were acquired from 230 nm-thick cross-sections using a Tecnai FEG 20 (FEI) microscope operated at 200 kV. Images were collected at a nominal magnification of 7800×, providing a pixel size of 2.8 nm. Alignments and reconstructions were performed using the IMOD software (https://bio3d.colorado.edu/imod/) (48). For volume EM, samples were prepared as previously described (46), and images were acquired using a FEG-SEM Quanta 250 (FEI) equipped with a 3View-system (Gatan, Inc). The image acquisition was performed with a pixel size of 5.5 nm, cutting depth of 50 nm, beam voltage of 2.5 kV, spot size of 3, and Torr pressure of 0.2. Segmentation and visualization were performed using Microscopy Image Browser (MIB, (49)) and Amira (FEI VSG), respectively.

Distances between intercellular spaces were measured from TEM images using Fiji/ImageJ (NIH). For unbiased quantification, an evenly spaced grid was placed over the TEM images, and the width of perpendicularly oriented clear intercellular junctions crossing the grid lines (0.5 μm × 0.5 μm) was measured along the whole plasma membranes (46). The dimensions of lamellipodia-like protrusions were measured from volume EM data using the MIB software (http://mib.helsinki.fi/) by applying the 3D lines tool and grid lines of 200 nm distance.

For protein immunolocalization, MDCK cells were prepared for immunolabeling as described previously (46). Briefly, cells grown in Transwell cell culture inserts were fixed with PLP fixative (2% formaldehyde, 0.01 M periodate, 0.075 M lysine–HCl in 0.075 M phosphate buffer, pH 7.4) (50) for 2 h at room temperature. Cells were then permeabilized with 0.01% saponin and immunolabeled using anti-HA antibody (1:60) and 1.4-nm gold particle–conjugated Fab fragments against mouse immunoglobulin G (Nanoprobes, 1:60). The nanogold was silver enhanced using an HQ Silver (Nanoprobes, #2012) for 4 to 5 min and was gold toned with 0.05% gold chloride. After silver enhancement, the sample was processed for plastic embedding as described above.

### BioID

BioID was performed with biological replicates twice according to the previously described protocol (33, 51). For stable transfection, MDCK cells were transfected with either myc-BirA (control) or full-length MTSS1 containing a C-terminal myc-BirA plasmid with the neomycin resistance gene (*neo*) for G418 (Geneticin) resistance. The established stable cell lines were incubated for 24 h in complete media supplemented with 50 μM biotin. After three PBS washes, the cells were collected and analyzed by mass spectrometry (MS). The samples were reserved for Western blotting and immunofluorescence.

The protein-protein interaction network was analyzed using stringApp in Cytoscape. Protein complexes in the protein-protein interaction network were identified using MCODE in Cytoscape. Functional analysis of the identified protein–protein interactions was performed using PANTHER.

### Statistical analysis

Statistical significance was determined using the nonparametric Mann–Whitney U test/Wilcoxon rank-sum test. The significance level, alpha, was set at 0.05.

Box plots were generated with BoxPlotR. Center lines show medians, box limits indicate 25th and 75th percentiles as determined using R software, and whiskers extend 1.5 times the interquartile range from the 25th and 75th percentiles (52).

## Data availability

All data are contained within the manuscript.

## Ethics approval

This article does not contain any studies with human participants or animals.

## Supporting information

This article contains supporting information (53).

## Conflict of interest

The authors declare that they have no conflicts of interest with the contents of this article.
