# Supplementary material for: Actin-rich lamellipodia-like protrusions contribute to the integrity of epithelial cell–cell junctions
Source: J Biol Chem. 2023 Mar 3;299(5):104571. doi: 10.1016/j.jbc.2023.104571 (PMC10173786; doi:10.1016/j.jbc.2023.104571)
Supplement: Supporting Information [file mmc10.docx]

**Supporting Information**

**Actin-rich lamellipodia-like protrusions contribute to the integrity of epithelial cell–cell junctions**

**Yosuke Senju^1,2,*^, Toiba Mushtaq^1^, Helena Vihinen^1^, Aki Manninen^3^, Juha Saarikangas^4,5^, Katharina Ven^1^, Ulrike Engel^6^, Markku Varjosalo^1^, Eija Jokitalo^1^, Pekka Lappalainen^1^**

1. Helsinki Institute of Life Science (HiLIFE) - Institute of Biotechnology, University of Helsinki, Helsinki, Finland

2. Research Institute for Interdisciplinary Science (RIIS), Okayama University, Okayama, Japan

3. Faculty of Biochemistry and Molecular Medicine, Biocenter Oulu, University of Oulu, Oulu, Finland

4. Helsinki Institute of Life Science (HiLIFE), University of Helsinki, Helsinki, Finland

5. Molecular and Integrative Biosciences Research Programme, Faculty of Biological and Environmental Sciences, Neuroscience Center, University of Helsinki, Helsinki, Finland.

6. Nikon Imaging Center and Centre for Organismal Studies, Heidelberg University, Heidelberg, Germany

* For correspondence: Yosuke Senju, yosuke.senju@okayama-u.ac.jp.

**Figure S1. MTSS1 and PI(4,5)P_2_ localize and cluster at adherens junctions in polarized MDCK cells**

(A) Immunofluorescence co-localization of E-cadherin with MTSS1 and F-actin at adherens junctions in polarized MDCK cells stably-expressing HA-tagged MTSS1. F-actin was labeled with Alexa Fluor 488 phalloidin (Scale bar: 10 µm).

(B) Pearson correlation coefficient (right) between MTSS1 and F-actin fluorescence intensities along the boxed region in the model diagram at adherens junctions in Fig. 1C. A Pearson correlation coefficient value close to 1 indicates that the line profiles of fluorescence intensities of MTSS1, and either F-actin or myosin II overlap each other (as depicted on the right side of the boxplot), and thus co-localize (co-cluster) at adherens junctions. A Pearson correlation coefficient value close to -1 indicates that the line profiles of fluorescence intensities of MTSS1, and either F-actin or myosin II do not overlap each other (as depicted on the right side of the boxplot). Center lines show medians; box limits indicate the 25th and 75th percentiles as determined by R software; whiskers extend 1.5 times the interquartile range from the 25th and 75th percentiles. n = 21. Note that a value close to 1 for the Pearson correlation coefficient between MTSS1 and F-actin indicates that MTSS1 may thus promote F-actin assembly at cell–cell junctions.

(C) Pearson correlation coefficient (right) between MTSS1 and myosin II fluorescence intensities along the boxed region in the model diagram across adherens junctions in Fig. 1D. n = 30.

**Figure S2. MTSS1 and non-muscle myosin II do not precisely co-localize with each other at cell**–**cell junctions.**

(A) Immunofluorescence images of MDCK cells stably expressing HA-tagged MTSS1. Non-muscle myosin IIA was visualized using anti-non-muscle myosin heavy chain II-A antibody and F-actin with fluorescent phalloidin (Scale bar: 10 µm).

(B) Magnification (from the region indicated by boxes in panels A), shown as composite. Orthogonal y-z and x-z projections along the yellow lines in the upper panel demonstrate that, although both non-muscle myosin IIA and MTSS1 localize to adherens junctions, non-muscle myosin IIA is positioned further away from the intercellular junction compared to MTSS1 (Scale bar: 2 µm).

(C) A 3D-SIM super-resolution image of F-actin in MDCK cells, in which axial position is color encoded. F-actin was stained with Alexa Fluor 488 phalloidin. In the enlarged view, the lamellipodia-like membrane protrusions and stress fiber-like actin bundles at adherens junctions are indicated by arrowheads and arrows, respectively (Scale bar: 5 µm).

(D) EGFP-tagged PLCdelta-PH domain labeling one of the phosphoinositides, PI(4,5)P_2_, localized at cell–cell junctions in a confluent monolayer. The normalized fluorescence intensity along the cell–cell junctions indicates that PI(4,5)P_2_ clusters in the same way as MTSS1 and F-actin at adherens junctions (Scale bar: 10 µm).

**Figure S3**. **The WAVE2 complex is important for the membrane and actin dynamics at cell–cell junctions**

(A) Fluorescence intensities of WAVE2, MTSS1, and F-actin from a line-scan along one representative cell–cell junctions in the boxed region in the model diagram (left) indicate that all three proteins co-localize in clusters at adherens junctions (middle). Pearson correlation coefficients (right) between WAVE2 and MTSS1/F-actin fluorescence intensities along the line profiles at adherens junctions. When the Pearson correlation coefficient value is close to 1, WAVE2, MTSS1, and F-actin co-localize (co-cluster) at adherens junctions. MTSS1 may thus promote F-actin assembly at adherens junctions upstream of WAVE2:Arp2/3 pathway. n = 37 (MTSS1) and 37 (F-actin).

(B-D) Representative images of confluent MDCK cells stably expressing HA-tagged MTSS1 and stained for endogenous WAVE2. F-actin was visualized using fluorescent phalloidin (Scale bar: 10 µm).

(E) Magnification (from the region indicated by boxes in panels B and C) shown as composite, and as orthogonal y-z and x-z projections along the yellow lines in the upper panel (Scale bar: 2 µm).

**Figure S4. Protein complexes in the protein-protein interaction network identified using BioID.**

(A-C) Putative interaction partners of MTSS1 at adherens junctions in polarized MDCK cells stably expressing BirA-tagged MTSS1 were identified using proteomics approach, BioID. Immunofluorescence staining of co-localizations of MTSS1 with the ENAH-VASP-RAPH1 complex at adherens junctions were observed by confocal microscopy (Scale bar: 10 µm).

**Figure S5**. **Dynamics of the plasma membrane protrusions at cell**–**cell junctions**

(A) Polarized MDCK cells were treated with DMSO (control), Arp2/3 complex specific inhibitor CK-666, or myosin II specific inhibitor (-)-blebbistatin, and F-actin was visualized with Alexa Fluor 488 phalloidin (left). The ratios (junctional/cytoplasmic) of the F-actin fluorescence intensities at adherens junctions in MDCK cells treated with DMSO (control), CK-666, or (-)-blebbistatin (right). Diminished F-actin localization to adherens junctions was observed in both Arp2/3- and myosin II –inhibitor treated cells. n = 31 (DMSO), 31 (CK-666), and 35 (Blebbistatin) (Scale bar: 10 µm).

(B) F-actin fluorescence intensities along line profiles at adherens junctions (exemplified in the boxed region in the model diagram on the right) indicate that actin-rich clusters are largely absent from Arp2/3-inctivated cells, and that F-actin is more uniformly distributed at adherens junctions. Relatively straight adherens junctions were chosen to facilitate line profile analysis.

(C) Kymographs along line profiles at cell–cell junctions in Figure 5B were also generated in DMSO- (control) or CK-666-treated MDCK cells expressing EGFP-tagged LifeAct (a marker to visualize F-actin) in a confluent monolayer. Note that F-actin clusters are persistent and laterally immobile. However, actin in the clusters turn over more rapidly (see FRAP analysis in Figure 5C). F-actin clusters are not prominent at cell–cell junctions in CK-666-treated cells (Scale bar: 10 µm).

**Figure S6. *Wasf2* knockout in MDCK cells**

(A and B) Sanger sequencing of genomic regions surrounding the target sequences in exon 3 and exon 4 of the *Wasf2* gene from the wild-type (WT) and two *Wasf2* knockout (KO) clones. sgRNAs were designed to target exons 3 and 4 of the *Wasf2* gene (highlighted in orange). Insertion of a single nucleotide (highlighted in blue) resulted in a translational frameshift in exon 4, whereas the exon 3 KO clone contained variants. Next Generation Sequencing (NGS) of the exon 3 KO clone revealed two KO variants, a short (56.2%) and a long (43.4%) variant. Mutations in the exon 3 KO clone led to two truncated WAVE2 proteins (77 amino acids long, 8.62 kDa size and 87 amino acids long, 9.89 kDa size) due to premature stop codons (-). Mutation in the exon 4 KO clone led to a truncated WAVE2 protein (111 amino acids long, 12.56 kDa size) due to a premature stop codon (-).

(C) *Wasf2* KO by CRISPR-Cas9 in MDCK cells was confirmed using western blotting (WB). Despite having a calculated molecular weight (MW) of ~54 kDa, the band ran at ~80 kDa, which is higher than the calculated MW. This may be due to the proline rich residues in the WAVE2 sequence (53). Tubulin was used as loading control to normalize that the protein loading was the same.

**Figure S7. *Wasf2* KO cells display diminished F-actin intensity at adherens junctions.**

(A) Immunofluorescence images showing WAVE2, β-catenin, and F-actin staining in a co-culture of WT and *Wasf2* KO MDCK cells. In addition to junctional WAVE2, β-catenin, and F-actin, the basal F-actin is shown (Scale bar: 10 µm).

(B) Magnification (from the region indicated by boxes in panel A) shown as composite, and as orthogonal y-z and x-z projections along the yellow lines in the upper panel (Scale bar: 2 µm).

**Movie S1-S3**

Electron tomography of cell–cell junctions in polarized MDCK cells.

**Movie S4**

The 3View for serial block face imaging of the 3D ultrastructures of cell–cell junctions in polarized MDCK cells.

**Movie S5**

Live imaging of F-actin in lamellipodia-like membrane protrusions at cell–cell junctions in confluent MDCK cells.

**Movie S6**

Live imaging of MTSS1 in lamellipodia-like membrane protrusions at cell–cell junctions in confluent MDCK cells.

**Movie S7**

Live imaging of lamellipodia-like membrane protrusions at cell–cell junctions in confluent MDCK cells co-expressing EGFP-tagged MTSS1 and mCherry-tagged LifeAct, which labels F-actin.

**Movie S8**

Live imaging of EGFP-tagged LifeAct (a marker to visualize F-actin) in lamellipodia-like membrane protrusions at cell–cell junctions in confluent MDCK cells treated with DMSO (control).

**Movie S9**

Live imaging of EGFP-tagged LifeAct (a marker to visualize F-actin) in lamellipodia-like membrane protrusions at cell–cell junctions in confluent MDCK cells treated with the Arp2/3 complex-specific inhibitor CK-666.

**References**

53. C. Yan, *et al.*, WAVE2 deficiency reveals distinct roles in embryogenesis and Rac-mediated actin-based motility. *EMBO J* **22**, 3602–3612 (2003).
