# Supplementary figures and images for: Actin-rich lamellipodia-like protrusions contribute to the integrity of epithelial cell–cell junctions
Source: J Biol Chem. 2023 Mar 3;299(5):104571. doi: 10.1016/j.jbc.2023.104571 (PMC10173786; doi:10.1016/j.jbc.2023.104571)

**A**

E-cadherin

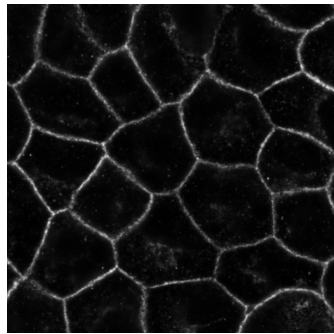

F-actin

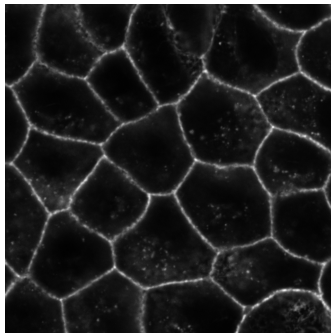

MTSS1

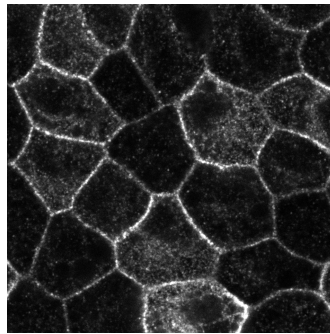**B**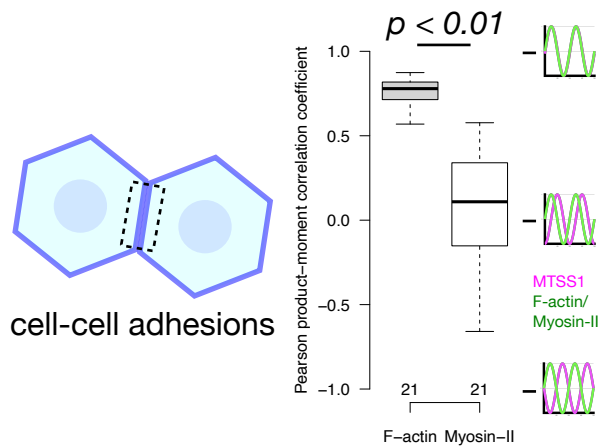**C**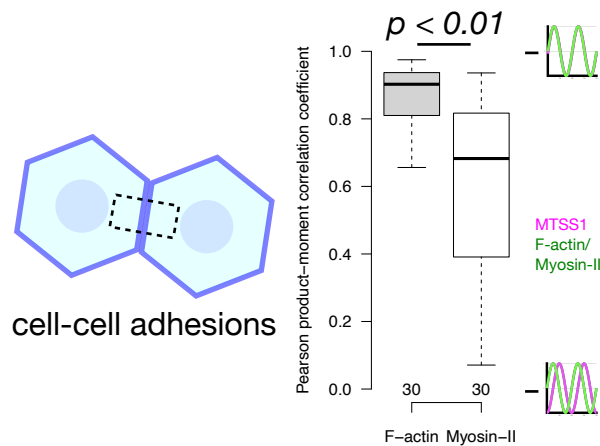

Supplement: Supporting Figure S1 [file mmc11.pdf]

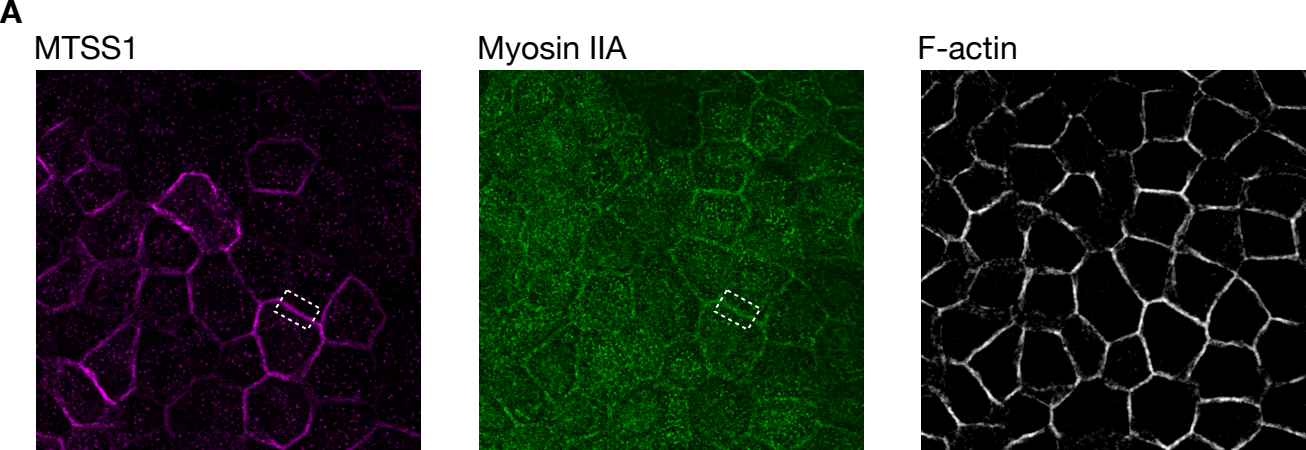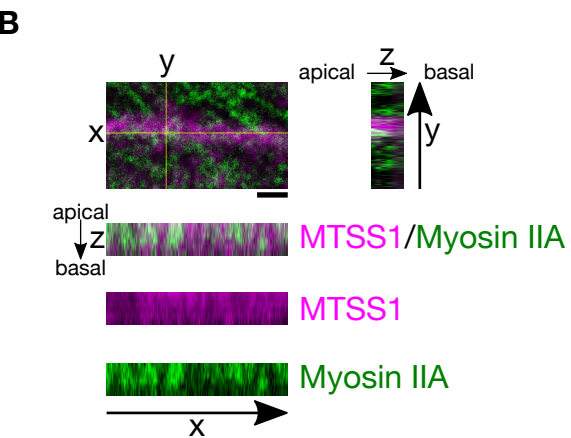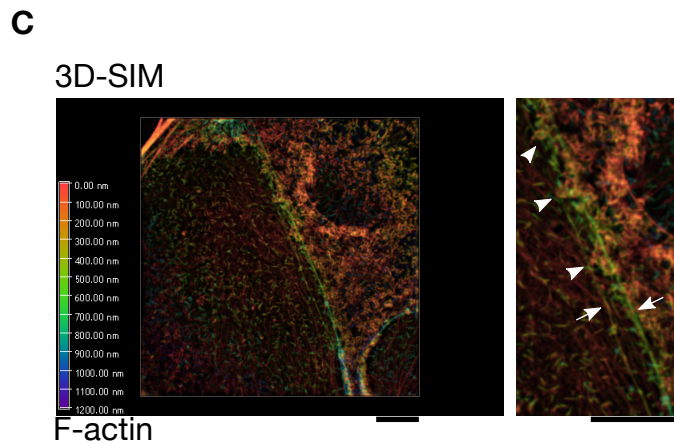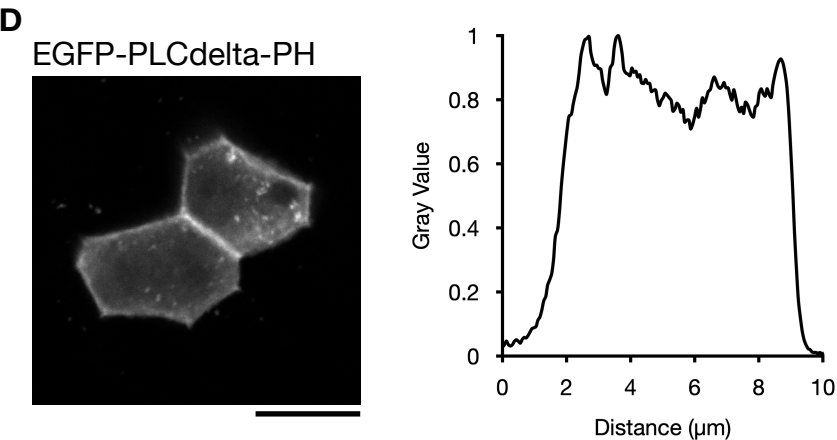

Supplement: Supporting Figure S2 [file mmc12.pdf]

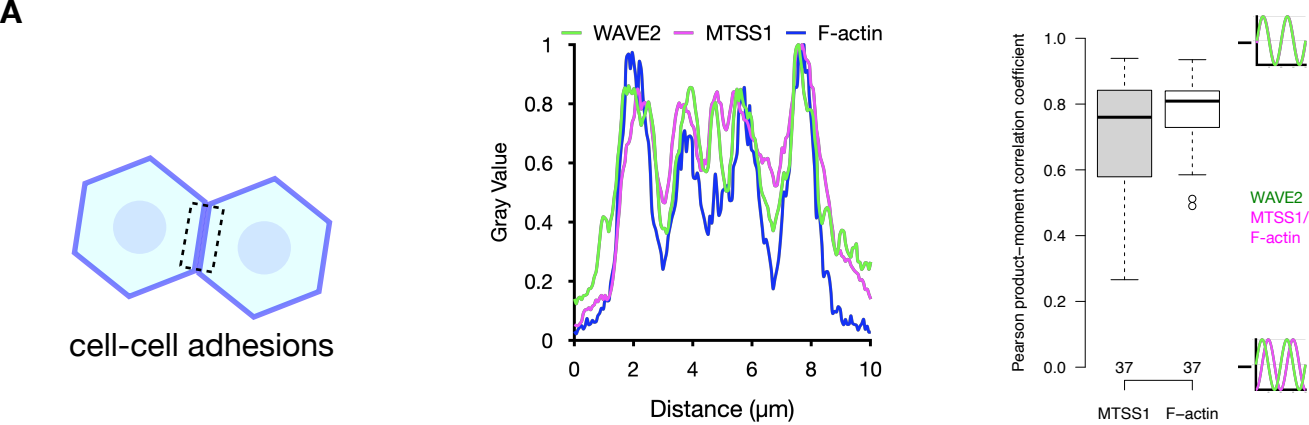

**B** MTSS1

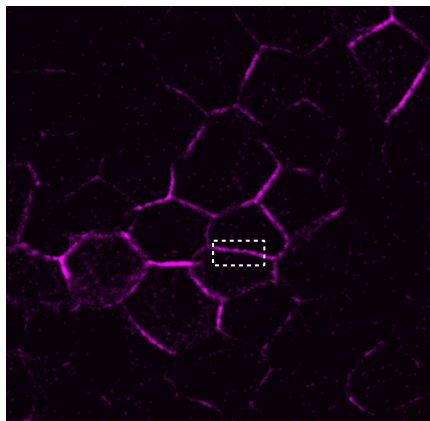

**C** WAVE2

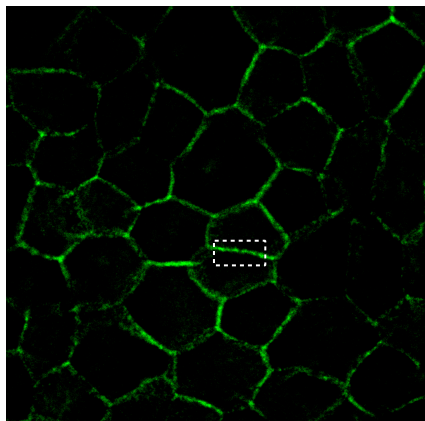

**D** F-actin

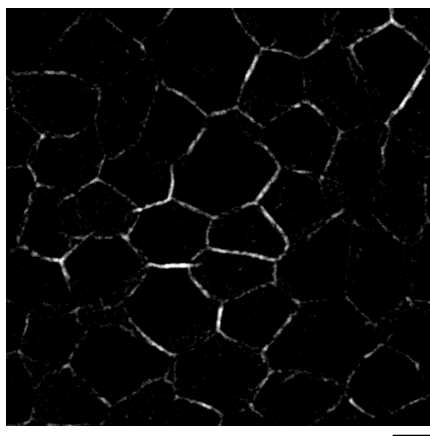

**E**

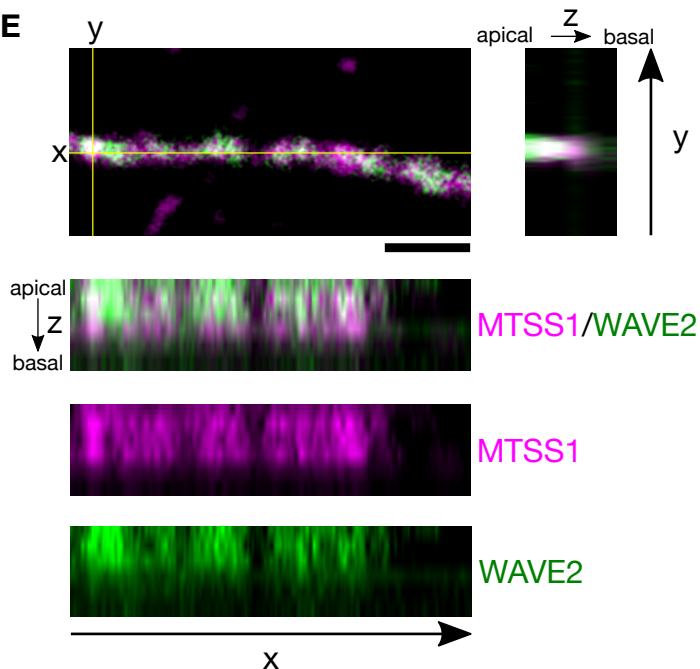

Supplement: Supporting Figure S3 [file mmc13.pdf]

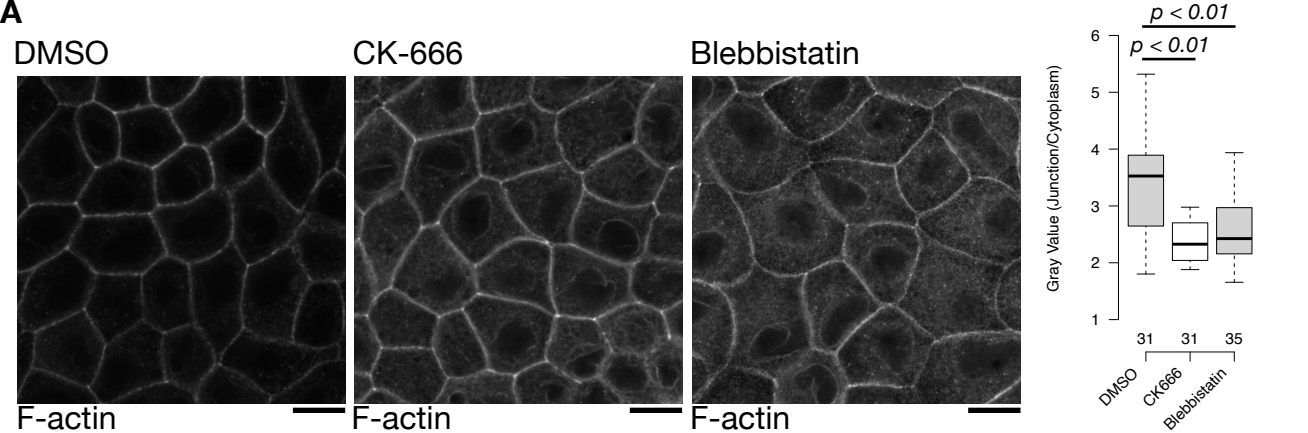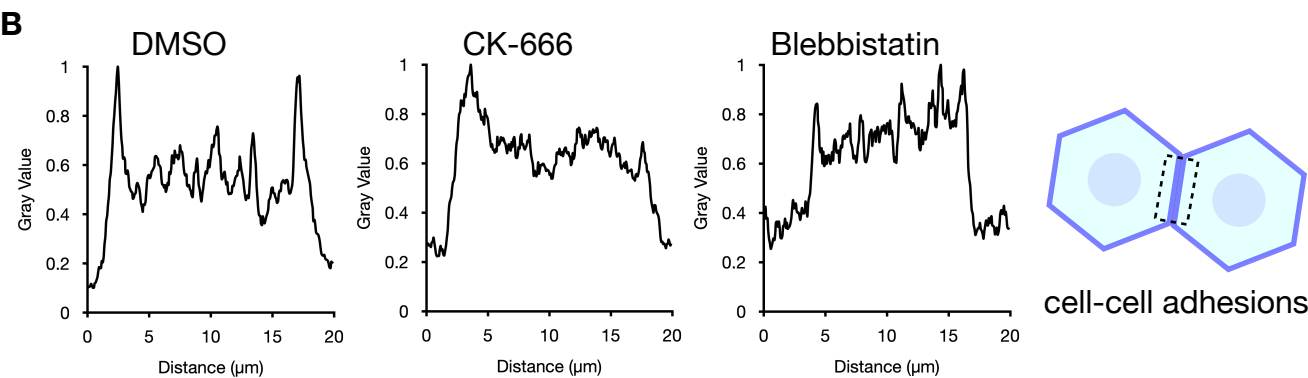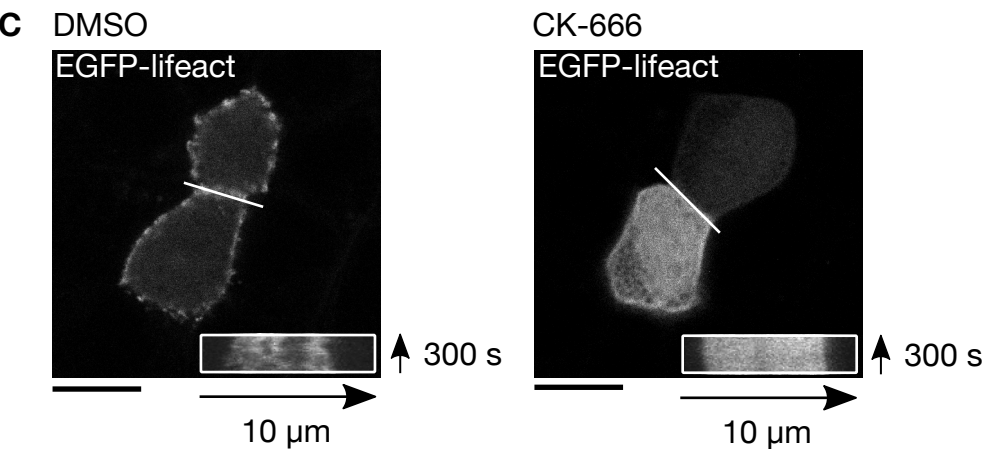

Supplement: Supporting Figure S5 [file mmc15.pdf]

**A**

WAVE2

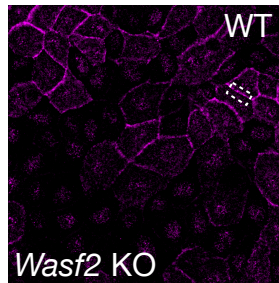 $\beta$ -Catenin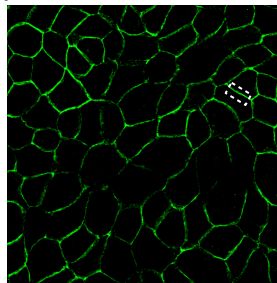

F-actin (lateral)

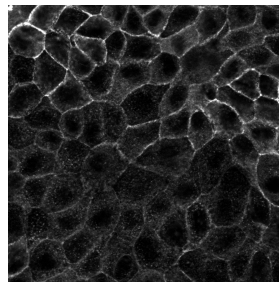

F-actin (basal)

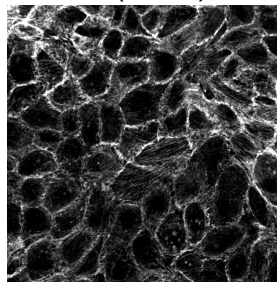**B**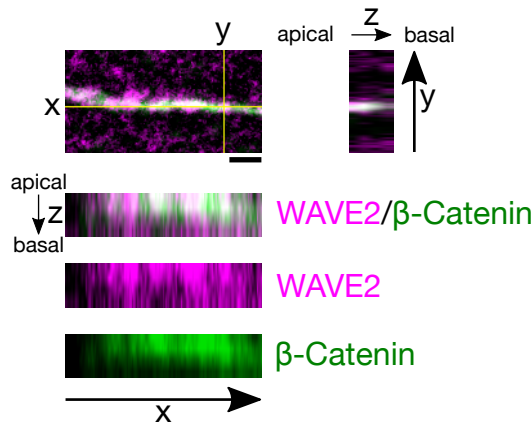

Supplement: Supporting Figure S7 [file mmc17.pdf]
