# Supplementary material for: Actin-rich lamellipodia-like protrusions contribute to the integrity of epithelial cell–cell junctions
Source: J Biol Chem. 2023 Mar 3;299(5):104571. doi: 10.1016/j.jbc.2023.104571 (PMC10173786; doi:10.1016/j.jbc.2023.104571)
Supplement: Supporting Figure S4 [file mmc14.pdf]

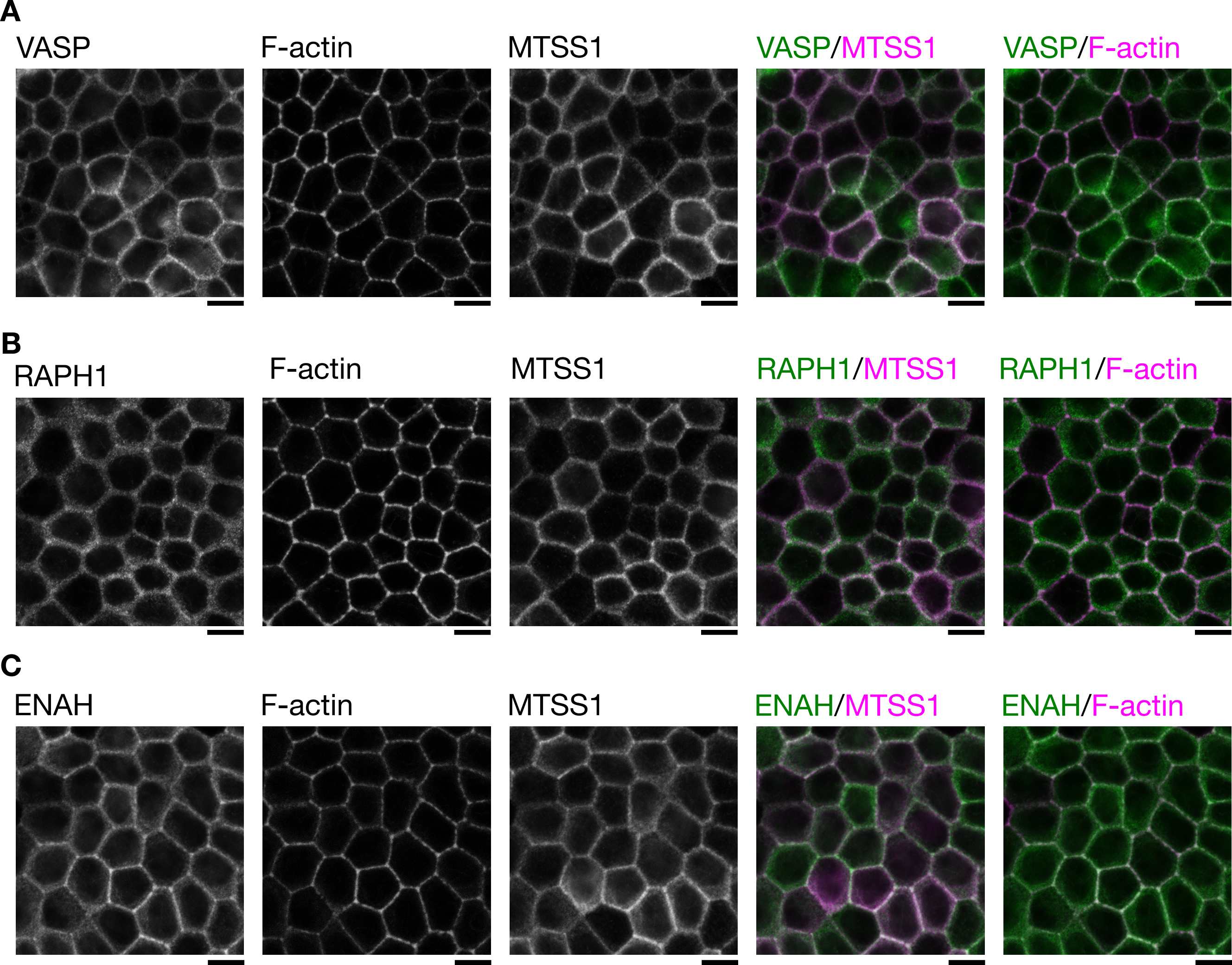
A
VASP
F-actin
MTSS1
VASP/MTSS1
VASP/F-actin
B
RAPH1
F-actin
MTSS1
RAPH1/MTSS1
RAPH1/F-actin
C
ENAH
F-actin
MTSS1
ENAH/MTSS1
ENAH/F-actin
