# Supplementary material for: Actin-rich lamellipodia-like protrusions contribute to the integrity of epithelial cell–cell junctions
Source: J Biol Chem. 2023 Mar 3;299(5):104571. doi: 10.1016/j.jbc.2023.104571 (PMC10173786; doi:10.1016/j.jbc.2023.104571)

A WAVE2 exon 3

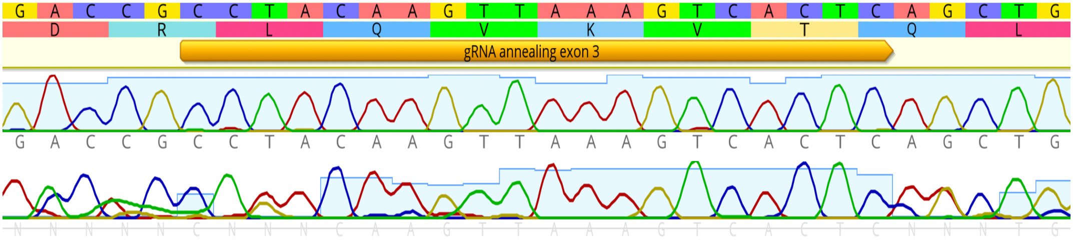

Nucleotide sequence

Ref GACCGCCTACAAGTTAAAGTCACTCAGCTG
WT GACCGCCTACAAGTTAAAGTCACTCAGCTG
KO1 GACCGCCTAACAAGTTAAAGTCACTCAGCTG
KO2 GACCGCCTTTTTGACCGCCTTGCTGAGAGGGTCGACCTAACTTTTTGACAAGTTAAAGTCACTCAGCTG

Protein sequence

Ref DRLQVKVTQLDPKEEEVSLQGINT
WT DRLQVKVTQLDPKEEEVSLQGINT
KO1 DRLTS-
KO2 DRLFDRLAERVDLTF-

B WAVE2 exon 4

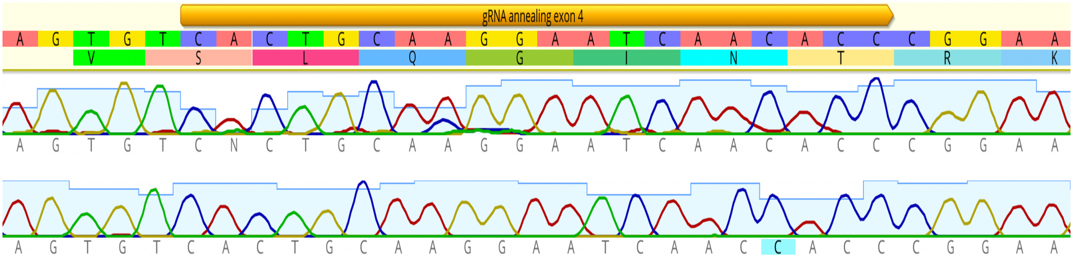

Nucleotide sequence

Ref AGTGTCACTGCAAGGAATCAACACCCGGAA
WT AGTGTCNCTGCAAGGAATCAACACCCGGAA
KO AGTGTCACTGCAAGGAATCAACCACCCGGAA

Protein sequence

Ref VSLQGINTRKAFRSSTIQDQKLFD
WT VSLQGINTRKAFRSSTIQDQKLFD
KO VSLQGINHPEGLQKLYHSRPEAF-

C

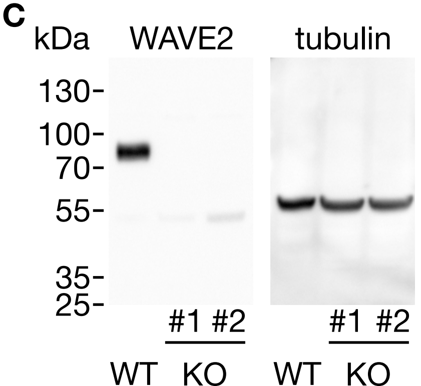

Supplement: Supporting Figure S6 [file mmc16.pdf]
